# Intraoperative Ultrasound Staging for Colorectal Liver Metastases in the Era of Liver-Specific Magnetic Resonance Imaging: Is It Still Worthwhile?

**DOI:** 10.1155/2019/1369274

**Published:** 2019-09-22

**Authors:** Serena Langella, Francesco Ardito, Nadia Russolillo, Elena Panettieri, Serena Perotti, Caterina Mele, Felice Giuliante, Alessandro Ferrero

**Affiliations:** ^1^Department of General and Oncological Surgery, Ospedale Mauriziano “Umberto I”, Torino, Italy; ^2^Unit of Hepatobiliary Surgery, Fondazione Policlinico Universitario A. Gemelli IRCCS, Università Cattolica Del Sacro Cuore, Rome, Italy

## Abstract

**Background:**

To assess the efficacy of intraoperative ultrasound (IOUS) compared with liver-specific magnetic resonance imaging (MRI) in patients with colorectal liver metastases (CRLMs).

**Methods:**

From January 2010 to December 2017, 721 patients underwent MRI as a part of preoperative workup within 1 month before hepatectomy and were considered for the study. Early intrahepatic recurrence (relapse at cut surface excluded) was assessed 6 months after the resection and was considered as residual disease undetected by IOUS and/or MRI. IOUS and MRI performance was compared on a patient-by-patient basis. Long-term results were also studied.

**Results:**

A total of 2845 CRLMs were detected by MRI, and the median number of CRLMs per patient was 2 (1–31). Preoperative chemotherapy was administered in 489 patients (67.8%). In 177 patients, 379 new nodules were intraoperatively found and resected. Among 379 newly identified nodules, 317 were histologically proven CRLMs (11.1% of entire series). The median size of new CRLMs was 6 ± 2.5 mm. Relationships between intrahepatic vessels and tumors differed between IOUS and MRI in 128 patients (17.7%). The preoperative surgical plan was intraoperatively changed for 171 patients (23.7%). Overall, early intrahepatic recurrence occurred in 8.7% of cases. To assess the diagnostic performance, 24 (3.3%) recurrences at the cut surface were excluded; thus, 5.4% of early relapses were considered for analysis. The sensitivity of IOUS was superior to MRI (94.5% vs 75.1%), while the specificity was similar (95.7% vs 95.9%). Multivariate analysis at the hepatic dome or subglissonian and mucinous histology revealed predictive factors of metastases missing at MRI. The 5-year OS (52.1% vs 37.8%, *p*=0.006) and DF survival (45.1% vs 33%, *p*=0.002) were significantly worse among patients with new CRLMs than without.

**Conclusions:**

IOUS improves staging in patients undergoing resection for CRLMs even in the era of liver-specific MRI. Intraoperative detection of new CRLMs negatively affects oncologic outcomes.

## 1. Introduction

Various imaging modalities have been developed in the field of liver surgery for accurate detection of colorectal metastases (CRLMs) [1]. Nevertheless, additional CRLMs can be found at the time of surgery in up to 25% of patients [2–9]. We previously reported [9] that intraoperative ultrasonography (IOUS) enabled detection of 17.6% of new nodules in patients undergoing resection for CRLMs. In this series, we also demonstrated that IOUS provides significant information about vascular relationships between tumors and hepatic vessels. Therefore, surgical plan was modified according to IOUS findings in 24.6% of cases. The published data on the impact of intraoperative staging are extremely heterogeneous because of differences among centers in preoperative diagnostic workup and surgical policies. Moreover, magnetic resonance imaging (MRI) with liver-specific contrast agent has dramatically improved the sensitivity of detection of liver tumors [10, 11]. Although the efficacy of this new imaging modality to stage hepatic disease in patients with CRLMs has been reported in several studies [12, 13], whether IOUS can improve liver staging when MRI is performed as a part of preoperative workup remains unclear. The aim of this study is to assess the efficacy of IOUS compared with liver-specific MRI in patients undergoing hepatectomy for CRLMs.

## 2. Materials and Methods

Between January 2010 and December 2017, 721 consecutive patients who underwent liver resection for CRLMs at two institutions (Ospedale Mauriziano, Torino, and Policlinico Gemelli, Roma, Italy) were considered for the study. Eligibility criteria were one- or two-stage resection for CRLMs (with or without preoperative chemotherapy), age ≥18 years, written informed consent, preoperative MRI with liver-specific contrast agent performed within 1 month before hepatectomy, IOUS accomplished by surgeon during the procedure, postoperative follow-up at least 6 months.

Data from prospectively collected databases were retrospectively reviewed. The collection and registration of the original database were performed according to regulations and with the approval of the institutional review boards of the two hospitals.

Primary endpoint was to compare diagnostic performance of MRI and IOUS to stage intrahepatic disease. The performances of IOUS and MRI were also compared in patients who did not receive preoperative chemotherapy. Secondly, we evaluated the impact of new CRLMs intraoperatively found on long-term outcomes.

### 2.1. Preoperative Workup

At diagnosis, all patients were evaluated with computed tomography (CT) scans and MRI. CT scans were performed with a multislice helical CT using a 3 mm collimation and reconstruction at 1 and 2.5 mm. Images were acquired using a triphasic hepatic protocol following a noncontrast evaluation of the liver. Images were obtained 11, 80, and 180 seconds after the start of intravenous injection of iopromide (Ultravist® 370, Bayer HealthCare Pharmaceuticals Inc., Wayne, NJ) at a rate of 3.5 mL/s. MRI was conducted on a 1.5 T superconducting system using a liver-specific contrast agent (EOB-gadoxetic acid disodium, Primovist, Bayer Schering Pharma AG, Berlin, Germany). All MR images were preoperatively evaluated by radiologists skilled in liver pathology and diffusion-weighted imaging (DWI). Fluorodeoxyglucose positron emission tomography was also performed in selected cases. After chemotherapy, restaging was accomplished by MRI and thoracic CT scans or thoracoabdominal CT scans in the presence of extrahepatic disease. Chemotherapy response was assessed by using the Response Evaluation Criteria in Solid Tumors (RECIST) [14].

### 2.2. Intraoperative Staging

Abdominal exploration and intraoperative liver ultrasonography (Aloka Prosound Alpha 7 with 7.5 MHz intraoperative miniconvex probe, Aloka Co., Tokyo, Japan; Philips HDI® 5000 SonoCT with 8 MHz to 4 MHz intraoperative convex probe ATL Entos CT8-4, Koninklijke Philips Electronics, Eindhoven, Netherlands) were always performed as the first step to assess the site, extent of the disease, and the tumor's relationships with major intrahepatic vessels and to define the extension of the required resection. The surgeon conducted IOUS for all patients according to a standardized protocol. A similar technique was used for laparoscopic liver ultrasound. This was performed with a multifrequency (5–10 MHz) flexible linear-array laparoscopic transducer (UST-5536-7.5; Hitachi Aloka Medical) and a Pro Focus 2202 Ultrasound System with Laparoscopic Transducer Type 8666-RF (Bk Medical, Herlev, Denmark).

Contrast-enhanced intraoperative ultrasound (CEIOUS) was additionally performed in selected cases. CEIOUS was achieved with a convex 2–6 MHz harmonic frequency transducer. In all patients, 2.4 mL sulfur hexafluoride microbubbles (SonoVue®, Bracco Imaging, Milan, Italy) was injected intravenously through a peripheral vein by the anesthesiologist.

All nodules consistent with CRLMs found intraoperatively by IOUS and/or CEIOUS that were not detected at preoperative MRI were classified as new lesions. During surgery, MR images were always available on a computer screen, which allowed a real-time comparison with intraoperative findings. In patients who underwent chemotherapy, disappeared liver metastases (DLMs) on preoperative MRI that were detected intraoperatively were not considered new lesions.

### 2.3. Histopathologic Examination

The pathologist was informed about the site of preoperatively detected CRLMs and new nodules. Specimens were fixed, embedded in paraffin, and stained with hematoxylin-eosin. Then, 0.5 cm slices were taken for microscopic examination. Steatosis was estimated as the percentage of involved hepatocytes and categorized as defined by Kleiner et al. [15]: no fatty change (<5%), mild (5% to <33%), moderate (33% to <66%), or severe (≥66%).

### 2.4. Diagnostic Performance Analysis

We conducted patient-by-patient analysis to evaluate MRI and intraoperative staging performance (IOUS and CEIOUS). Early intrahepatic recurrences were registered at 6 months after the resection and were considered residual disease undetected by intraoperative staging and/or MRI (false negative: FN). Recurrences were assessed using radiological imaging during the follow-up. Patients were evaluated every 3 months with physical examination, measurement of CEA levels, and abdominal ultrasonography or thoracoabdominal CT. Local recurrence on the cut liver surface was not considered an FN. In patient-by-patient analysis, sensitivity was defined as the number of patients without FN lesions divided by the total number of patients. Conversely, specificity was defined as the number of patients without false-positive (FP) lesions divided by the total number of patients. By definition, we considered the positive and negative predictive values (PPV and NPV, respectively) as the proportions of positive and negative results in true-positive and true-negative results. The likelihood ratio was calculated for both positive (LR+, likelihood ratio positive: sensibility/1−specificity) and negative (LR−, likelihood ratio negative: 1−sensibility/specificity) results.

### 2.5. Definitions

  Indirect signs to identify liver metastases by IOUS were presence of bile duct dilatation, distortion, or interruption of the venous wall.
  Types of hepatectomies were classified according to the Brisbane 2000 terminology [16].
  Were considered mucinous CRLMs, those histologically proven liver metastases comprising more than 50% mucinous carcinoma.
  Local recurrence was defined as intrahepatic relapse at cut surface of the previous hepatectomy.
  Subglissonian metastasis was defined as lesions within 1 cm of the liver surface.

### 2.6. Statistical Analysis

All statistical analyses were performed using IBM SPSS software (v20.1). The distribution of variables was analyzed using the Kolmogorov–Smirnov test. Categorical variables were compared using the chi-square test, Fisher's exact test, or Pearson's test as appropriate. Continuous variables were compared between groups using the unpaired *t*-test or Mann–Whitney *U* test, as appropriate. Continuous variables were presented as median ± standard deviation (SD) or range. Categorical variables were represented as number and percentage in brackets. Diagnostic performance was evaluated assessing sensitivity, specificity, PPV, NPV, and likelihood ratio. Cohen's kappa coefficient was used to assess the interrater reliability of preoperative and intraoperative imaging. The results have been interpreted as follows: values ≤0 as indicating no agreement and 0.01–0.20 as none to slight, 0.21–0.40 as fair, 0.41–0.60 as moderate, 0.61–0.80 as substantial, and 0.81–1.00 as almost perfect agreement. Uni- and multivariate binary logistic regression analyses were performed to assess the predictive factors for missing CRLMs at MRI. After univariate analysis, a *p* value ≤0.05 was considered to include variables in the multivariate analysis. Receiver operating characteristic curves were plotted to identify the value of preoperative number of metastases and median number of neoadjuvant chemotherapy cycle in predicting missing CRLMs at MRI with a high sensitivity and specificity. Disease-free survival was measured from the date of hepatic resection until the date of radiographic detection of recurrence, death, or last follow-up. Overall survival was measured from the date of hepatic resection until the date of death or last follow-up. The Kaplan–Meier method was used to estimate survival probabilities, which were compared using the log-rank test. All *p* values were two sided, and *p* ≤ 0.05 was considered statistically significant.

## 3. Results

Patients were investigated with a median of 3 (range 2–4) preoperative imaging modalities (US, CT scan, and MRI and PET). All patients were staged with MRI, and fluorodeoxyglucose positron emission tomography was undertaken in 317 (43.9%) patients. A total of 2845 CRLMs were detected preoperatively using MRI. The median number of CRLMs per patients was 2 (1–31). Multiple (more than 3) CRLMs were observed in 358 (49.6) patients. The median diameter was 24 ± 22.05 mm. In 56 (7.7%) patients, CRLMs were from mucinous cancer.

Preoperative chemotherapy was administered in 489 patients (67.8%). In this subgroup, DLMs were present in 52 of 489 (10.6%) patients. Hepatic resections were minor in 592 (82.1%) patients. Among these, multiple liver resections were required in 517 (87.3%) cases. Minor resections were distributed as follows: 459 (77.5%) nonanatomical, 60 (10.1%) anatomical, and 73 (12.3%) both anatomical and nonanatomical. Two stage procedures were accomplished in 30 (4.2%) patients. Redo-resection for recurred CRLMs was performed in 50 of 721 patients (6.9%). A laparoscopic approach was used in 103 (14.3%) patients to perform 11 (10.7%) major and 92 (89.3%) minor hepatectomies.

Preoperative and operative data are detailed in Table 1.

### 3.1. Intraoperative Findings and Management

In 177 patients, 379 (13.3%) new nodules were intraoperatively found and resected. Among 379 newly identified nodules, 317 (83.6%) were histologically proven CRLMs (11.1% of entire series). The 62 FP cases (16.4%) were classified by pathologists as hemangiomas (19), focal steatosis (14), biliary hamartoma (12), granulomatous inflammation (9), fibrosis (6), and focal nodular hyperplasia (2). Furthermore, 38 (73%) of 52 DLMs were found intraoperatively (not considered new CRLMs).

The liver was hyperechoic in half of the patients (363, 50.3%). The median new CRLM size was 6 ± 2.5 mm, and most were hypoechoic (77.3%). The new CRLMs were only rarely detected by indirect signs (3.8%) or CE-IOUS (5.9%). Features of new CRLMs were summarized in Table 2.

Seventy out of 317 (22%) new lesions were located at the hepatic dome (Segments (Sgs) 8 and 4a). The remaining new nodules were distributed as follows: nine in Sg 1 (2.8%), 31 in Sg 2 (9.7%), 38 in Sg 3 (11.9%), 41 in Sg 4b (12.9%), 43 in Sg 5 (13.5%), 45 in Sg 6 (14.2%), and 40 in Sg 7 (12.7%). Twenty-eight new CRLMs were sited within 1 cm from the liver surface.

Vascular relationships between intrahepatic vessels and tumors differed between IOUS and MRI in 128 patients (17.7%). In 31 (4.3%) patients, 46 (1.6%) lesions suspected for metastases at preoperative imaging were not identified or assessed intraoperatively as metastases. Among the 46 lesions left in situ because of IOUS findings, only two were subsequently diagnosed as metastases during the follow-up (rate of FN lesion 4.3%). Overall, in 171 (23.7%) patients, the preoperative surgical plan changed according to intraoperative findings. Commonly, in case of new CRLMs, limited additional resections were required (83%).

Overall, 232 (32.2%) patients were scheduled for upfront surgery (without preoperative chemotherapy). Among these, MRI preoperatively identified 504 CRLMs, and the median number of CRLMs per patient was 1 (1–4). In this subset of patients, IOUS detected 68 (13.5%) histologically proven CRLMs in 31 patients (13.3%). IOUS also revealed 5 (0.9%) new nodules in 4 (1.7%) patients that—after resection—were classified by the pathologist as benign lesions (FP). On the other hand, all but one CRLM identified at MRI were confirmed intraoperatively. The median size of new CRLMs was 5 mm (1–7). CRLMs newly identified were mainly hypoechoic (27/31, 87%). Features of new CRLMs are summarized in Table 2.

### 3.2. Diagnostic Performance Analysis

Performance of MRI and intraoperative staging was compared on a patient-by-patient basis.

Overall early intrahepatic recurrences were 8.7%. To assess the diagnostic performance, 24 (3.3%) recurrences at the cut surface were excluded; thus, 5.4% of early relapses were considered for analysis. According to the rates of FP and FN patients, IOUS was more sensitive than MRI (94.5% vs 75.1%), while the specificity was similar (95.7% vs 95.9%). PPV of MRI was 79.7% vs 93.5% of IOUS while NPV was 95.7% (MRI) vs 96.5% (IOUS). The LR− was 0.26 (MRI) vs 0.07 (IOUS), and the LR+ was 17.5 (MRI) vs 21.9 (IOUS). Finally, Cohen's kappa coefficient was 0.73, indicating a substantial agreement between MRI and IOUS.

Diagnostic performance was also assessed among patients who did not receive preoperative chemotherapy. In this subset of patients, IOUS revealed a higher sensitivity (MRI 84%, IOUS 97.4%). Both sensitivity and sensibility (MRI 99.5%, IOUS 98.3%) were improved compared to those of the whole population with a substantial agreement between MRI and IOUS (Cohen's kappa coefficient = 0.80). All parameters considered for diagnostic performance analysis are detailed in Table 3.

### 3.3. Predictor of Missing CRLMs at MRI

We assessed predictors of missing CRLMs at MRI. Receiver operating characteristic curve analysis revealed a significant predictive value of the median number of preoperative chemotherapy cycles (area under the curve 0.605; *p* < 0.001, *n* = 5 cycles, sensitivity 61.3%, and specificity 53.7%) and median number of metastases at preoperative imaging (area under curve 0.624; *p* < 0.001, *n* = 3 cycles, sensitivity 58.5%, and specificity 60%) for missing colorectal metastases at MRI.

Univariate analysis showed an increased risk to miss CRLMs at MRI among male patients (*p*=0.011) and those who received preoperative chemotherapy (*p*=0.002), particularly related to an irinotecan-based regimen (*p*=0.001) or association with biologic agents (*p* < 0.001) and to the number of cycles administered (*p* < 0.001). MRI also more frequently missed CRLMs in cases of multiple (>3) lesions, metastases from mucinous tumors (*p* < 0.001), and nodules located at the hepatic dome or subglissonian (*p* < 0.001). In multivariate analysis, only location at the hepatic dome or subglissonian and mucinous histology resulted in predictive factors of missing metastases at MRI (*p* < 0.001). Results of uni- and multivariate analyses are reported in Table 4.

### 3.4. Intraoperative Staging and Long-Term Outcomes

Overall, 487 patients experienced recurrence after resection, whereas the liver was the site of relapse in 199 cases. Intrahepatic recurrences were significantly more frequent in patients with new CRLMs than without (36.2 vs 26.8%, *p*=0.027). The R1 resection rate was 26.0% in patients with new CRLMs and 27.0% in patients without new CRLMs (*p*=0.834). Adjuvant chemotherapy was administered similarly among patients with or without new CRLMs (66.4% vs 67.1%, *p*=0.768). Among 721 patients, 3 postoperative deaths were excluded from survival analysis. Two-stage hepatectomy (*n* = 8) was considered part of one procedure. Finally, 710 patients were considered for analysis.

**Figure 1:**
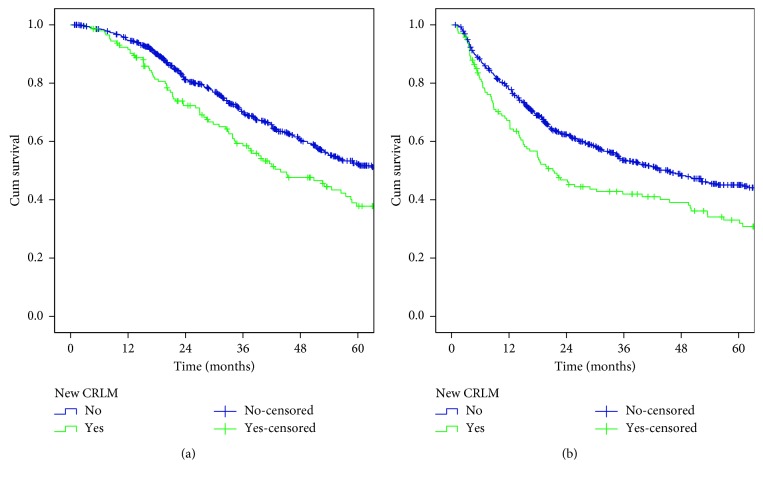
Overall (a) and disease-free (b) survival rates after hepatectomy. Comparison between patients with new CRLMs (green line) and without (blue line).

The 5-year OS (52.1% vs 37.8%, *p*=0.006) and DF survival (45.1% vs 33%, *p*=0.002) were significantly worse among patients with new CRLMs than without (Figures 1(a) and 1(b)).

## 4. Discussion

Nowadays, MRI with liver-specific agents is the most accurate radiologic imaging modality to stage hepatic disease in patients with CRLMs [10, 14]. As suggested by several studies, the evaluating hepatocyte-specific uptake enables accurate detection and characterization of CRLMs [11]. Furthermore, DWI allows an evaluation of changes in the diffusion properties of water molecules in tissues, which adds useful information to conventional imaging sequences [17]. These advantages of MRI are maintained even after chemotherapy. Macera et al. [18] demonstrated that combining DWI with gadoxetic agent-enhanced MRI (EOB-MRI) significantly increased the diagnostic accuracy (89.2%, 95%CI 83.4–93.4) and sensitivity (91%, 95%CI 85.1–95.1) in patients with CRLMs with preoperative chemotherapy; this was particularly effective in the detection of small lesions (<1 cm). In a randomized trial [13], comparison of the total number of CRLM detected at initial imaging *versus* the number found intraoperatively and at pathological examination of resected specimens showed the greatest number of patients with equal assessments (88%) in the EOB-MRI group (compared with 74% and 62% in conventional MRI and CT scan groups, respectively). Diagnostic confidence was high or very high for 98.3% of patients with EOB-MRI; consequently, surgical plans were less frequently changed during surgery (28%) compared with patients staged by conventional MRI (32%) or CT scan (47%).

Considering the improved performance of MRI over time, it can be assumed that intraoperative staging has a limited value at present. In the present study, the diagnostic performance of IOUS was superior to MRI. New histologically proven CRLMs were found in 11.1% of patients. The high rate of FN explains the low sensitivity of MRI. However, in patient-by-patient analysis, specificity remains high for both MRI and IOUS. This reflects the low FP rate for both techniques, which enable adequate characterization of liver nodules in most cases. In 2013, we demonstrated [9] that IOUS showed the best diagnostic performance compared with CT scan, PET, and MRI. We assessed the performance of staging techniques comparing the pre- and intraoperative findings with the results of pathological examination and early intrahepatic recurrence at 6 months after surgery as an indicator of residual disease. This parameter is a good marker for FN for both preoperative staging and intraoperative staging, and it allows precise evaluation of the drawbacks of IOUS. Therefore, this methodological approach was also applied in the present series.

It is well known that preoperative chemotherapy may negatively affect the accuracy of preoperative staging and intraoperative staging [19]. This is mainly due to the chemotherapy-related changes in liver parenchyma and modification of CRLM features. After chemotherapy, MRI with liver-specific contrast agents guarantees the most accurate preoperative staging of hepatic disease compared to other imaging modalities [20]. However, some small CRLMs may disappear at preoperative MRI after chemotherapy [21]. In this series, vanishing metastases at preoperative MRI were excluded from the analysis to avoid a possible overestimation of new CRLMs intraoperatively found. Moreover, to overcome the potential bias related to the chemotherapy administration, we also evaluated the diagnostic performance of MRI and IOUS in patients who underwent liver resection without preoperative chemotherapy. In this subset of patients, sensibility and sensitivity of both MRI and IOUS were improved. Nevertheless, IOUS assured the higher sensitivity rate.

To depict the pitfalls in the MRI staging, we assessed the predictors of “missing” metastases at MRI. Multivariate analysis demonstrated an increased risk of missing CRLMs at MRI in the case of subglissonian nodules or located at hepatic dome, such as for metastases from mucinous tumors. The liver surface can be better assessed intraoperatively [5, 18, 22, 23]; moreover, the evaluation of the hepatic dome during MRI may be limited by artefacts related to respiratory movement. Furthermore, mucinous tumors could mimic benign lesions, worsening the diagnostic accuracy, particularly in the case of small nodules [24, 25]. In agreement with previous studies [4, 9], we confirmed that newly identified CRLMs are more likely to be hypoechoic. Because of steatosis or postchemotherapy changes in the hepatic parenchyma, patients who underwent hepatectomy for CRLMs often present a “bright” liver. This condition enables detection of hypoechoic nodules and reduces the value of CE-IOUS staging because the liver is naturally enhanced [2]. These findings could explain the high accuracy of IOUS reported in the present series, even if CE-IOUS was not performed systematically.

Several surgical series [2–9] have reported the superiority of IOUS to stage hepatic disease in CRLMs compared with various imaging modalities. The improvements of imaging modalities over the years represent a challenge for the current role of IOUS. As expected, the rate of new CRLMs found by IOUS decreased in the most recent series but remains noteworthy (ranging from 8% to 21%). Unfortunately, results from published studies cannot be generalized because of extreme variability related to the different preoperative workup and technological progress over time. Notably, the present study considered many patients resected in recent years at two tertiary centers. This population is homogeneous, and the process of MRI with liver-specific contrast agents was similar for all patients. Previous published data also showed that the impact on management is extremely heterogeneous and it is strongly affected by surgical policies. Our centers shared a parenchymal sparing philosophy to face CRLMs; this explains the significant impact of intraoperative findings on changing surgical plans.

In patients with HCC, the intraoperative detection of new tumors negatively affects oncologic outcomes [26]. However, the impact of new CRLMs on long-term outcomes has been poorly evaluated. In the present study, we showed that hepatic recurrences are significantly more frequent among patients with newly identified CRLMs and they present worse OS and DF survival. We previously demonstrated that additional CRLMs are more likely to be found in patients with more aggressive disease. We therefore suggest considering the new CRLMs during postoperative decision making along with other known prognostic factors. For example, this subset of patients may benefit from adjuvant chemotherapy to reduce the risk of relapse. Moreover, because the possibility of reresection for recurred CRLMs can significantly improve survival [27], we also suggest a strict postoperative surveillance to detect and manage hepatic recurrences as early as possible.

This study presents some limitations, mainly related to its retrospective nature. Even if both IOUS and RM were performed by surgeons and radiologists skilled in the field of liver malignancies, different physicians were involved in the study. Nevertheless, this is the largest series to date focusing on this topic and comparing the performance of MRI with liver-specific contrast agents to IOUS.

In conclusion, IOUS improves staging in patients undergoing resection for CRLMs even in the era of liver-specific MRI. Intraoperative detection of new CRLMs negatively affects oncologic outcomes.

## Figures and Tables

**Table 1:** Preoperative and operative characteristics of 721 patients with 2845 CRLMs preoperatively assessed by MRI (whole population) and 232 patients who did not receive preoperative chemotherapy.

Characteristics	Whole population *n* = 721	Patients without chemotherapy *n* = 232
Age (years), median ± SD	64 ± 10.8	66 ± 10.0
Male, *n* (%)	453 (62.2)	149 (64.2)
BMI (kg/m^2^), median ± SD	25 ± 3.34	27 ± 4.0
Preoperative chemotherapy	489 (67.8)	—
Number of cycles, median ± SD	5 ± 6.5	—
Oxaliplatin based, *n* (%)	358 (49.7)	—
Irinotecan based, *n* (%)	208 (28.8)	—
Biologics, *n* (%)	309 (42.9)	—
Response to chemotherapy^*∗*^		
PR	306 (62.6)	—
SD	154 (31.5)	—
PD	29 (5.9)	—
Preoperative radiologic workup		
PET total body, *n* (%)	317 (44)	107 (46.1)
Number of LMs, median (range)	2 (1–31)	1 (1–4)
Maximum diameter (mm), median ± SD	24 ± 22.05	19.4 ± 23.0
Types of resection		
(1) Minor hepatectomy, *n*(%)	592 (82.1)	194 (83.6)
(2) Laparoscopic resection, *n*(%)	103 (14.3)	62 (26.7)
(3) Redo-resection, *n*(%)	50 (6.9)	28 (18)

BMI, body mass index; PR, partial response; SD, stable disease; PD, progression disease. ^*∗*^Rates calculated on 489 patients who received preoperative chemotherapy.

**Table 2:** Details of new CRLMs intraoperatively found in the whole population and in patients who did not receive preoperative chemotherapy.

New CRLM Features	Whole population (*n* = 317)	Patients without chemotherapy (*n* = 31)
Diameter (mm), median ± SD	6 ± 2.5	5 ± 2.3
Number per patients, median (range)	1 (1–9)	1 (1–6)
US aspect		
Hypoechoic, *n* (%)	245 (77.3)	27 (87)
Hyperechoic, *n* (%)	46 (14.5)	1 (9.6)
Isoechoic, *n* (%)	11 (3.4)	1 (3.2)
Location subglissonian, *n* (%)	75 (23.6)	6 (19.3)
New CRLMs identified by CE-IOUS, *n* (%)	19 (5.9)	2 (6.4)
New CRLMs identified by indirect signs, *n* (%)	12 (3.8)	1 (3.2)

**Table 3:** Performance of intraoperative staging and MRI.

	Whole Population		No preop. chemotherapy	
	MRI	IOUS^1^	MRI	IOUS

Number of patients without FN lesions	541	682	195	226
Total number of patients	721	721	232	232
Sensitivity (%)	75.1	94.5	84.1	97.4
PPV (%)	79.7	93.5	83.7	97.8
LR−	0.26	0.07	0.16	0.03

	MRI	IOUS^1^	MRI	IOUS

Number of patients without FP lesions	692	690	231	228
Total of patients	721	721	232	232
Specificity (%)	95.9	95.7	99.5	98.3
NPV (%)	95.7	96.5	97.4	97.7
LR+	17.5	21.9	84	48.5

MRI, magnetic resonance imaging; FP, false positive; FN, false negative; PPV, positive predictive value; NPV, negative predictive value. ^1^IOUS with or without contrast enhancement.

**Table 4:** Univariate and multivariate analyses of factors predicting missing metastases at MRI.

	Univariate analysis		Multivariate analysis		
	Missing CRLMs (*n* = 146)	No missing CRLMs (*n* = 575)	*p*	OR (CI 95%)	*p*

Age (years)	63 ± 10.6	64 ± 10.8	0.742	—	
Sex male	105 (71.9)	348 (60.5)	0.011	n.s.	
BMI ≥30 kg/m^2^	24 (16.4)	85 (14.8)	0.618		
Chemotherapy regimen data					
Preoperative chemotherapy	115 (78.8)	374 (65.0)	0.002	n.s	
Oxaliplatin based	82 (56.2)	276 (48.0)	0.078	—	
Irinotecan based	58 (39.7)	150 (26.1)	0.001	n.s	
Oxaliplatin or irinoteca plus biologics^*∗*^	87 (59.6)	222 (38.6)	<0.001	n.s	
Number of cycles	6 ± 7.50	4 ± 5.66	<0.001	—	
Number of cycles >5	94 (64.4)	284 (49.4)	0.001	n.s	
Preoperative imaging data					
Number of CRLMs	4 ± 3.9	2 ± 4.2	<0.001	—	
Number of CRLMs >3	97 (66.4)	261 (45.4)	<0.001	n.s	
Diameter (mm)	20 ± 17.2	24 ± 23.0	0.103	—	
Location					
Subglissonian	25 (17.1)	3 (0.5)	<0.001	44.494 (11.785–167.977)	<0.001
Hepatic dome	60 (41)	10 (1.7)	<0.001	46.097 (20.911–101.617)	<0.001
Mucinous histology	42 (28.8)	14 (2.4)	<0.001	23.805 (11.173–50.719)	<0.001
Redo-resection	8 (5.5)	42 (7.3)	0.438	—	
Hepatic steatosis	111 (76.0)	396 (68.9)	0.091	—	
Mild	59 (40.4)	232 (40.3)	0.988	—	
Moderate	42 (28.7)	117 (20.3)	0.037	n.s.	
Severe	10 (6.8)	47 (8.1)	0.720	—	
No fatty	35 (23.9)	179 (31.1)	0.112	—	

Data are expressed as number (%) or median ± SD. BMI, body mass index; LM, liver metastasis; MRI, magnetic resonance imaging; IOUS, intraoperative ultrasonography. ^*∗*^Bevacizumab or cetuximab or panitumumab.

## Data Availability

The prospective data used to support the findings of this study are restricted by the ethics committee of Mauriziano Hospital in order to protect patient privacy. Data are available from corresponding author for researchers who meet the criteria for access to confidential data.
